# The efficacy of adding targeted agents to neoadjuvant therapy for locally advanced rectal cancer patients: a meta‐analysis

**DOI:** 10.1002/cam4.1298

**Published:** 2018-02-21

**Authors:** Xi Zhong, Zhonghua Wu, Peng Gao, Jinxin Shi, Jingxu Sun, Zhexu Guo, Zhenning Wang, Yongxi Song

**Affiliations:** ^1^ Department of Surgical Oncology and General Surgery The First Hospital of China Medical University 155 North Nanjing Street Heping District Shenyang 110001 China

**Keywords:** Efficacy, neoadjuvant therapy, pathologic complete response, rectal cancer, targeted agents

## Abstract

Patients with locally advanced rectal cancer (LARC) are at tremendous risk of metastatic diseases. To improve the prognoses of LARC patients, the efficacy of adding targeted agents to neoadjuvant therapy has been investigated by many researchers but remains controversial. A literature search of relevant databases was conducted through December 2016, 804 studies were identified and 32 investigations were ultimately included. A total of 1196 patients from 31 cohorts of 29 studies were eligible for quantitative synthesis in this single‐arm setting meta‐analysis. As pathologic complete response (pCR) shows promise as a prognosis indicator, we focused on pCR rates to evaluate whether adding targeted agents to neoadjuvant therapies improves the outcome of LARC patients. In our study, we revealed pooled estimates of pCR of 27% (95%CI, 21–34%) and 14% (95%CI, 9–21%) for bevacizumab‐relevant cohorts and cetuximab‐relevant cohorts, respectively. The safety of adding targeted agents to neoadjuvant therapy was also evaluated by pooling the data of Grade 3/4 toxicity. In conclusion, our study revealed that adding bevacizumab to the neoadjuvant therapy regimens provides appreciable pCR for LARC patients. Meanwhile, the efficacy of cetuximab remains inconclusive, RCTs with larger scale and better study design that stress more on mutational status are needed.

## Introduction

Rectal cancer is one of the most commonly diagnosed and deadliest cancers around the world 1. Patients with locally advanced rectal cancer (LARC) are at tremendous risk of metastatic diseases due to high rates of local and distant recurrence 2. In recent years, neoadjuvant chemoradiotherapy (nCRT) has proven its efficacy in tumor downstaging and local control 3, 4. Tumor downstaging, usually indicated by the endpoint of pathologic complete response (pCR) which is defined as the complete remission of tumor cells in the resected specimen, can increase the success of radical surgery, provide better opportunity for sphincter preservation, and may be associated with increased benefit from adjuvant therapy for LARC patients 4, 5, 6. Thus, nCRT followed by total mesorectal excision (TME) and adjuvant chemotherapy has been highly recommended in the National Comprehensive Cancer Network (NCCN) guidelines as a standard treatment for LARC patients 7. However, the pCR rates reported in many studies investigating the efficacy of nCRT are far from satisfying. The FFCD trial 8 showed a pCR rate of merely 11.4% for 375 patients in the nCRT arm, while only 13.7% of enrolled patients receiving nCRT reached pCR in the EORTC 22921 trial 9. pCR rates in other studies were also reported to be around 15% after the conduction of nCRT, indicating that improved nCRT regimens are necessary 10, 11, 12.

In the past decade, numerous emerging strategies for adding various targeted agents to nCRT regimens gained attention from oncologists. Targeted vascular endothelial growth factor (VEGF) inhibitors or epidermal growth factor receptor (EGFR) monoclonal antibodies such as bevacizumab, aflibercept, cetuximab, and panitumumab have been demonstrated to increase pCR rates and improve prognoses for metastatic colorectal cancer (mCRC) patients 13, 14, 15, 16. However, the NCCN recommends against the addition of bevacizumab, cetuximab, or panitumumab to nCRT regimens for resectable mCRC patients due to the higher incidences of wound‐healing complications, treatment‐related mortality, and reduced progression‐free survival (PFS) 7, 17, 18, 19, 20. On the contrary, targeted agents are recommended to be added to nCRT for unresectable mCRC patients despite the blurred standards for regimens 7.

In recent years, the efficacy of adding targeted agents to neoadjuvant therapies for LARC patients has been studied by abundant phase II trials, with pCR being the primary endpoint 21, 22, 23, 24. Yet, with few randomized controlled trials (RCTs) or clinical controlled trials (CCTs) available, we lack head‐to‐head data of time‐to‐event endpoints such as overall survival (OS) and PFS to evaluate the survival status of LARC patients receiving targeted agents in their nCRT regimens compared with those receiving nCRT alone. Thus, we focused on the pCR rates of LARC patients to study the efficacy of adding targeted agents to their neoadjuvant therapies. pCR has become a widely accepted prognostic indicator in LARC patients 25. Maas et al. 26. conducted a meta‐analysis of a large amount of individual patient data provided by 14 investigators and concluded that rectal cancer patients with pCR have better local control, a lower rate of distant recurrence, and improved survival compared to those without pCR. Several other investigations have also recommended pCR as an indicator of better outcome concerning local or distant recurrence, disease‐free survival (DFS), and OS 27, 28, 29, 30, 31. However, the reported pCR rates in the current studies vary, ranging from approximately 39.1% 32 to merely 4.3% 33. The sample sizes of these studies are also relatively small, the largest being 83^34^ and the smallest consisting of only eight patients 35. Therefore, the efficacy of adding targeted agents to the nCRT for LARC patients is still controversial.

Since pCR shows promise as a prognosis indicator, in this meta‐analysis we pooled the data of pCR rates extracted from the included studies to evaluate whether adding targeted agents to neoadjuvant therapies improves the outcome of LARC patients.

## Methods

### Study selection

This meta‐analysis was conducted following the Preferred Reporting Items for Systematic Reviews and Meta‐analysis (PRISMA) statements checklist 36.

The predefined criteria for eligible studies were as follow: (1) Patients with locally advanced rectal cancer (cT3‐4 primary rectal cancer and/or lymph node metastasis, without evidence of distant metastatic diseases). (2) Application of approved targeted agents in neoadjuvant therapy. (3) Endpoint of interest was pCR. (4) Original studies only (case reports, reviews, pooled‐analyses, and letters to the editor were excluded). Phase I clinical trials, which aim to evaluate the safety of novel agents, were also ruled out. (5) If investigations presented overlapping cohorts, studies which were more recently published and of higher quality were chosen.

### Search strategy

PubMed, Embase, and Web of Science were searched using a combination of the following terms: “rectal,” “rectum,” “colorectal,” “tumor,” “cancer,” “neoplasm,” “neoadjuvant,” “preoperative,” “perioperative,” “targeted,” “VEGF,” “EGFR,” “bevacizumab,” “cetuximab,” “C225,” “panitumumab,” “ramucirumab,” and “aflibercept” for relevant publications up to December 17, 2016. The references of the relevant studies were also screened for potential pertinent articles. There were no language restrictions used during the search.

### Data extraction

The primary endpoint was pCR and the second endpoint was the proportion of patients who encountered any Grade 3/4 toxic effects during preoperative chemoradiotherapy (preoperative Grade 3/4 toxicity). Data were manually extracted by two independent reviewers (X Zhong and Z.H. Wu) using standardized sheets. Any discrepancies between them were resolved by a third senior author.

The baseline details of the included studies were extracted by the same two reviewers and listed in the sheets mentioned above, and all the data entries were reviewed by the third senior author. The following data were extracted from VEGF‐inhibitor‐relevant studies: author and year of publication, study design, enrollment, regimen of neoadjuvant therapy, median age, tumor staging of included patients at enrollment, and the distance of primary tumor from anal verge. The following data were extracted from EGFR‐inhibitor‐relevant studies: author and year of publication, study design, enrollment, regimen of neoadjuvant therapy, median age, tumor staging of included patients at enrollment, the distance of primary tumor from anal verge, and KRAS status. The Newcastle‐Ottawa quality assessment scale (NOS) was applied to assess the quality of eligible studies for meta‐analysis 37. Studies which scored five or more were considered as moderate‐quality trials, whereas those with seven or more were regarded as high‐quality trials.

### Statistical analysis

All statistical analyses were performed using STATA version 12.0 (STATA, College Station, TX). Meta‐analyses were conducted by calculating the pooled estimates of pCR and preoperative Grade 3/4 toxicity, and a random‐effect model was used which provides more conservative estimates for the inevitable heterogeneity of included multicenter studies 38. To evaluate heterogeneity, the Cochrane's *Q* test and inconsistent index (I^2^) were performed, with I^2^ < 40% considered acceptable 19, 39, 40. Potential origins of heterogeneity were detected by performing sensitivity analysis. Publication biases were evaluated via funnel plots, Begg's funnel plot, and Egger linear regression test for further confirmation 19.

## Results

### Study selection and the characteristics of included studies

**Figure 1:**
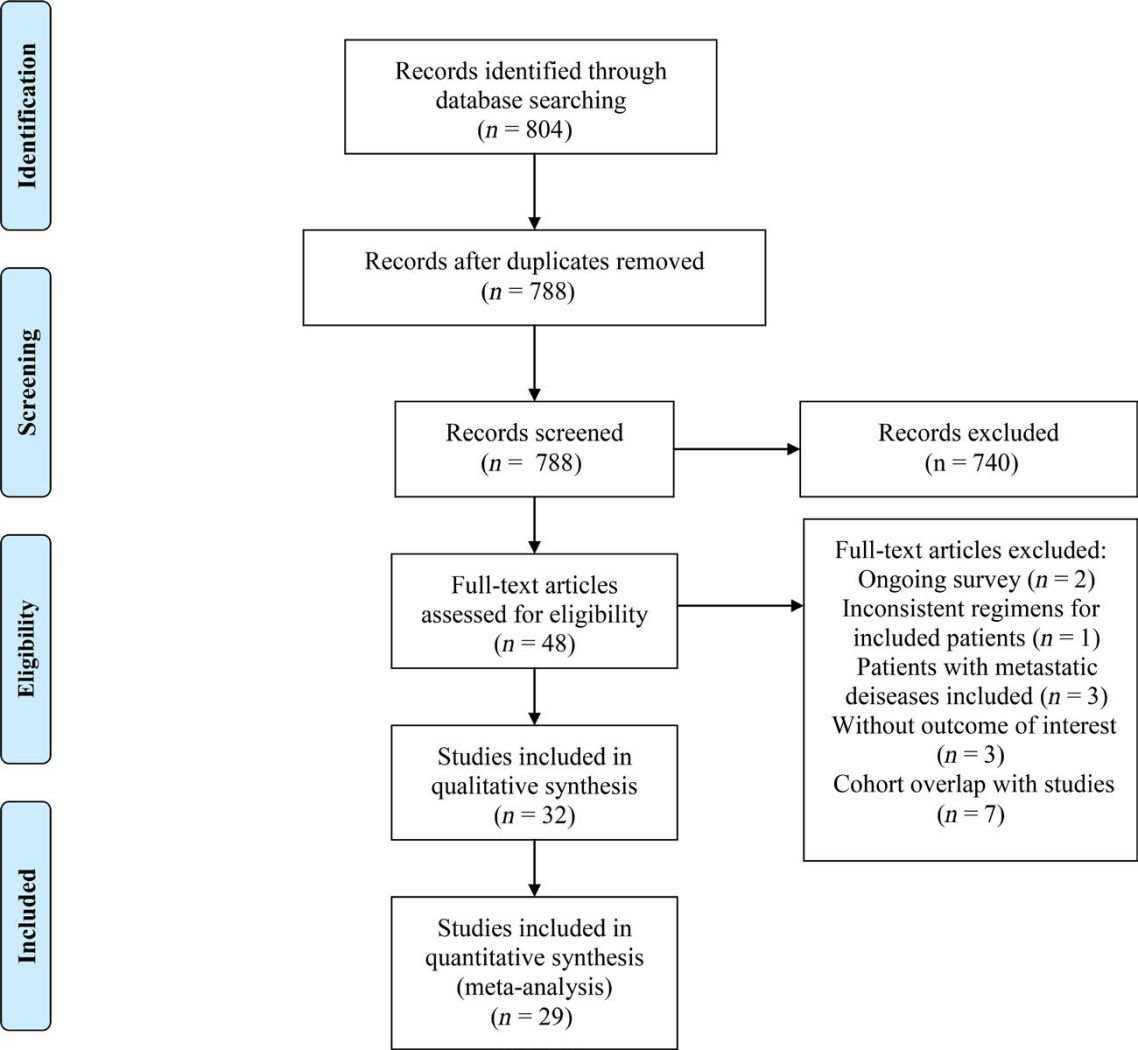
Selection of studies. Flow diagram showing the selection process for the included studies.

We identified 804 publications through the initial database search and screening the references of relevant studies, and 788 remained after removing duplicates. We excluded 740 records after reading their titles and abstracts, leaving 48 potentially eligible studies for full‐text review. A total of 32 studies were ultimately included, after ruling out 16 ineligible investigations which failed to meet the inclusion and exclusion criteria for this meta‐analysis. The included studies consisted of 21 for the VEGF inhibitor, bevacizumab 21, 22, 23, 32, 33, 35, 41, 42, 43, 44, 45, 46, 47, 48, 49, 50, 51, 52, 53, 54, 55, and 11 for EGFR inhibitors (eight for cetuximab 34, 56, 57, 58, 59, 60, 61, 62, one for nimotuzumab 63 and two for panitumumab 64, 65). These included one randomized clinical trial (RCT) 54 and three clinical controlled trials (CCT) 34, 53, 55, but we only analyzed cohorts which tested the addition of targeted agents to their neoadjuvant therapy regimens for this meta‐analysis. There were also two bevacizumab‐relevant studies 22, 52 consisting of two arms with bevacizumab in their neoadjuvant regimens, and we included all four cohorts for the meta‐analysis. Additionally, there was one study 49 consisting of two cohorts testing addition of bevacizumab, one in the neoadjuvant setting and the other in the postoperative setting, and we included only the former. The rest of the remaining studies were all single‐arm investigations. After the search, we determined that there were inadequate nimotuzumab‐relevant and panitumumab‐relevant studies to conduct a meta‐analysis. Thus, a total of 1196 subjects from 31 cohorts of 29 studies were eligible for quantitative synthesis. The whole selection process is presented in a flow diagram (Fig. 1). The baseline characteristics and data regarding the primary and secondary endpoints of the included studies for meta‐analysis are shown in Table 1 (bevacizumab‐relevant studies) and Table 2 (cetuximab‐relevant studies). The NOS quality assessment of the included investigations for meta‐analysis is shown in Table 3. Among the 29 studies, three scored seven points and were regarded as high‐quality studies and the remaining 26 all scored six points and were considered as studies of moderate quality.

**Table 1:** Baseline characteristics of cohort groups of bevacizumab for meta‐analysis

Study	Study design	Enrollment, *n*	Neoadjuvant therapy	Median age, year	Stage at enrollment, *n*	Distance from anal verge, cm	PreoperativeGrade3/4 toxicity	pCR
Blaszkowsky 2014	Prospective Phase I/II	32	5‐FU + Erlotinib + bevacizumab + RT	NR	cT3N0: 6; cT3N1: 15; cT3N2: 4; cT3Nx: 4; cT4N0: 2; cT4N1: 1	NR	46.9% (15/32)	33.3% (9/27)
Borg 2014	Prospective Phase II	46	Folfox‐4 + bevacizumab	60.6	cT3N0: 10; cT3N1: 31; Tc3N2: 5	NR	50% (23/46)	23.8% (10/42)
		45	5‐FU + bevacizumab + RT	60.1	cT3N0: 8; cT3N1: 28; cT3N2: 9	NR	20% (9/45)	11.4% (5/44)
Crane 2010	Prospective Phase II	25	Capecitabine + bevacizumab + RT	54.0	cT3N0: 5; cT3N0+: 20	≤5 cm: 15; >5 cm: 10	NR	32% (8/25)
Dellas 2013	Prospective Phase II	69	Capox + bevacizumab + RT	61.0	cT2Nx: 2; cT3N0: 12; cT3N0 + : 44; cT4N0: 3; cT4N+: 4:	5.92 ± 3.68 (Mean ± SD)	11.6% (8/69)	17.4% (12/69)
Dipetrillo2012	Prospective Phase II	25	mFOLFOX6 + bevacizumab + RT	50.0	T2: 2; T3: 20; T4: 3; N‐: 7; N+: 16; Nx: 2	NR	76% (19/25)	20% (5/25)
Fernandez‐Martos 2014	Prospective Phase II	46	Capox + bevacizumab	NR	cT3: 46	NR	NR	19.6% (9/46)
Garcia 2015	Prospective Phase II	41	Capecitabine + bevacizumab + RT	63.0	cT3^a^: 32; cT3a: 3; cT3b: 1; cT3c: 2; cT4: 2	NR	7.3% (3/41)	7.5% (3/40)
Gasparini 2012	Prospective Phase II	43	Capecitabine + bevacizumab + RT	64.0	cT2N1M0: 4; cT3N0M0: 14; cT3N1M0: 20; cT3NxM0: 1; cT4N1M0: 1; cT4N1M0: 1; cT4N2M0: 1; cTxN1M0: 1; cT4N2M1: 1	NR	NR	14.0%(6/43)
Hasegawa 2014	Prospective Pilot study	25	Capox + bevacizumab	63.0	cT4aN0M0: 1; cT4bN0M0: 3; cT2,cT3N2M0: 3; cT3,cT4aN1M0: 10; cT4aN2M0: 1; cT4bN1/N2M0: 7	5.0 (Median)	28% (7/25)	4.3% (1/23)
Landry 2015	Prospective Phase II	54	Capox + bevacizumab + RT	54.0	cT3: 50; cT4: 4; cNx: 2; cN0: 17; cN1: 30; cN2: 5	NR	NR	17.0% (9/53)
Nogue 2011	Prospective Phase II	47	Capox + bevacizumab + RT	58.5	cT3N0: 5; cT3N1: 22; cT3N2: 14; cT4N0: 2; cT4N1: 2; cT4N2: 2	NR	NR	35.6% (16/45)
Resch 2012	Prospective Phase II	8	Capecitabine + bevacizumab + RT	70.0	cT3: 8; cN0: 1; cN1: 4; cN2: 1; cNx: 2	NR	37.5% (3/8)	25% (2/8)
Sadahiro 2015	Prospective Phase II	52	S‐1 + bevacizumab + RT	59.0	cT2: 2; cT3: 49; cT4: 1; cN0: 16; cN1: 36	5.5 (Median)	1.9% (1/52)	19.2% (10/52)
Spigel 2012	Prospective Phase II	35	5‐FU + bevacizumab + RT	57.0	II: 11; III: 24	NR	NR	28.6% (10/35)
Uehara 2013	Prospective Phase II	32	Capox + bevacizumab	62.0	cT3: 13; cT4a: 9; cT4b: 10; cN0: 6; cN1: 14; cN2: 12	4.7 (Median)	25% (8/32)	13.3% (4/30)
Velenik 2011	Prospective Phase II	61	Capecitabine + bevacizumab + RT	60.0	cT3N0: 12; cT2N1: 1; cT3N1: 19; cT2N2: 2; cT3N2: 22; cT4N2: 5	6.0 (Median)	NR	13.3% (8/60)
Wang 2014	Prospective Phase II	12	FOLFOX + bevacizumab + RT/5‐FU + bevacizumab + RT	52.5	cT2: 1; cT3: 8; cT4: 3; cN0: 2; cN1: 2; cN2: 8	5 cm: 5; 5‐10 cm: 7; ≥10: 0	16.7% (2/12)	33.3% (4/12)
		6	FOLFOX + bevacizumab + RT	57.5	cT2: 0; cT3: 5; cT4: 1; cN0: 2; cN1: 4; cN2: 0	5 cm: 1; 5‐10 cm: 4; ≥10: 1	16.7% (1/6)	25% (1/4)
Xiao 2015	Prospective Phase II	25	5‐FU + oxaliplatin + bevacizumab + RT	45.0	cT2: 2; cT3: 9; cT4a: 8; cT4b: 6; cN‐: 4; cN+: 21	≤5 cm: 7; >5 cm: 18	NR	39.1% (9/23)
Koukourakis 2011	Prospective Phase II	19	Capecitabine + bevacizumab + RT	68.0	pT3: 19; pT4: 0; pN1: 12	NR	NR	36.8% (7/19)
Salazar 2015	Prospective Phase II	44	Capecitabine + bevacizumab + RT	64.0	II A: 6; II B: 1; III B: 18; III C: 19	6.5 (Median)	15.9% (7/44)	15.9% (7/44)
Willett 2010	Prospective Phase II	32	5‐FU + bevacizumab + RT	51.0	cT3: 28; cT4: 4; cN0: 9; cN1‐2: 23	NR	21.9% (7/32)	15.6% (5/32)

pCR, pathologic complete response; RT: radiotherapy; 5‐FU, fluorouracil; FOLFOX, leucovorin plus fluorouracil plus oxaliplatin; Capox, capecitabine plus oxaliplatin; S‐1, tegafur plus gimeracil plus potassium oxonate; NR, not reported.

a It was not specified if the cT3 status was cT3a, cT3b or cT3c.

**Table 2:** Baseline characteristics of cohort groups of cetuximab for meta‐analysis

Study	Study design	Enrollment	Neoadjuvant therapy	Median age, year	Stage at enrollment	Distance from anal verge, cm	KRAS status	Preoperative Grade3/4 toxicity	pCR
Bengala 2009	Prospective Phase II	40	5‐FU + cetuximab + RT	61	uT3N0: 12; uT3N1: 25; uT4N1: 3	NR	Wild‐type: 30; Mutated: 9	NR	7.7% (3/39)
Horisberger 2009	Prospective Phase II	50	Capecitabine+Irinotecan+cetuximab+RT	57	cT2: 5; T3: 42; cT4: 2; Local relapse: 1; cN0: 13; cN+: 37	7.5 (Median) (1–13, Range)	NR	NR	8% (4/50)
Kim 2011	Prospective Phase II	40	CapIri + cetuximab + RT	56.5	cT3N0: 6; cT3N+: 30; cT4N0: 2; cT4N+: 2	≤5: 19 > 5: 21 5.5 (Median) (0–8.0, Range)	Wild‐type: 33; Mutated: 5	17.9% (7/39)	23.1% (9/39)
Machiels 2007	Prospective Phase I/II	40	Capecitabine + cetuximab + RT	61	cT2N+: 2; cT3N0: 18; cT3N+: 13; cT4N0: 5; cT4N+: 2	<6 cm: 25 6–10 cm: 10 > 10 cm: 5	NR	NR	5% (2/40)
Rodel 2008	Prospective Phase I/II	60	Capox + cetuximab + RT	61.5	cT2N1‐2: 1; cT3N0: 7; cT3N1‐2: 43; cT4N0: 2; cT4N1‐2: 7	7 ± 3.5 (Mean ± SD) 0–14 (Range) Lower third (≤6 cm): 27 Middle third (6–12 cm): 27 Upper third (≥12 cm): 6	NR	NR	8.9% (4/45)
Sun 2012	Prospective Phase II	63	Capecitabine + cetuximab + RT	64	cT3N0: 8; cT3N1: 21; cT3N2: 26; cT4N0: 2; cT4N1: 2; cT4N2: 4	5 (Median) (1–9, Range)	Wild‐type: 44; Mutated: 19	NR	12.7% (8/63)
Velenik 2012	Prospective Phase II	47	Capecitabine + cetuximab + RT	55	cT3N0: 3; cT2N1: 1; cT3N1: 13; cT2N2: 1; cT3N2: 15; cT4N2: 4	6 (Median) (1–11, Range)	Wild‐type: 30; Mutated: 7	NR	8.1% (3/37)
Dewdney 2012	Prospective Phase II	83	Capecitabine + cetuximab + RT	61	cT3c‐ T3d: 47; T4: 21	NR	aWild‐type: 46; Mutated: 37	NR	18% (15/83)

pCR, pathologic complete response; RT, radiotherapy; 5‐FU, fluorouracil; CapIri, capecitabine plus irinotecan; Capox, capecitabine plus oxaliplatin; NR, not reported.

a KRAS/BRAF status.

**Table 3:** The NOS quality of included studies

Study	Selection				Comparability		Outcome			Total	Quality
	REC	SNEC	AE	DO	SC	AF	AO	FU	AFU		
Blaszkowsky 2014	1	0	1	1	0	0	1	1	1	6	Moderate
Borg 2014	1	1	1	1	0	0	1	1	1	7	High
Crane 2010	1	0	1	1	0	0	1	1	1	6	Moderate
Dellas 2013	1	0	1	1	0	0	1	1	1	6	Moderate
Dipetrillo 2012	1	0	1	1	0	0	1	1	1	6	Moderate
Fernandez‐Martos 2014	1	0	1	1	0	0	1	1	1	6	Moderate
Garcia 2015	1	0	1	1	0	0	1	1	1	6	Moderate
Gasparini 2012	1	0	1	1	0	0	1	1	1	6	Moderate
Hasegawa 2014	1	0	1	1	0	0	1	1	1	6	Moderate
Landry 2015	1	0	1	1	0	0	1	1	1	6	Moderate
Nogue 2011	1	0	1	1	0	0	1	1	1	6	Moderate
Resch 2012	1	0	1	1	0	0	1	1	1	6	Moderate
Sadahiro 2015	1	0	1	1	0	0	1	1	1	6	Moderate
Spigel 2012	1	0	1	1	0	0	1	1	1	6	Moderate
Uehara 2013	1	0	1	1	0	0	1	1	1	6	Moderate
Velenik 2011	1	0	1	1	0	0	1	1	1	6	Moderate
Wang 2014	1	0	1	1	0	0	1	1	1	6	Moderate
Xiao 2015	1	0	1	1	0	0	1	1	1	6	Moderate
Koukourakis 2011	1	0	1	1	0	0	1	1	1	6	Moderate
Salazar 2015	1	1	1	1	0	0	1	1	1	7	High
Willett 2010	1	0	1	1	0	0	1	1	1	6	Moderate
Bengala 2009	1	0	1	1	0	0	1	1	1	6	Moderate
Horisberger 2009	1	0	1	1	0	0	1	1	1	6	Moderate
Kim 2011	1	0	1	1	0	0	1	1	1	6	Moderate
Machiels 2007	1	0	1	1	0	0	1	1	1	6	Moderate
Rodel 2008	1	0	1	1	0	0	1	1	1	6	Moderate
Sun 2012	1	0	1	1	0	0	1	1	1	6	Moderate
Velenik 2012	1	0	1	1	0	0	1	1	1	6	Moderate
Dewdney 2012	1	1	1	1	0	0	1	1	1	7	High

REC, representativeness of the exposed cohort; SNEC, selection of the nonexposed cohort; AE, ascertainment of exposure; DO, demonstration that outcome of interest was not present at start of study; SC, study controls for age, sex; AF, study controls for any additional factors; AO, assessment of outcome; FU: follow‐up long enough (36M) for outcomes to occur; AFU, adequacy of follow‐up of cohorts (≥90%). “1″ means that the study satisfies the item and “0” means the opposite situation.

### The efficacy and safety of VEGF inhibitor

**Figure 2:**
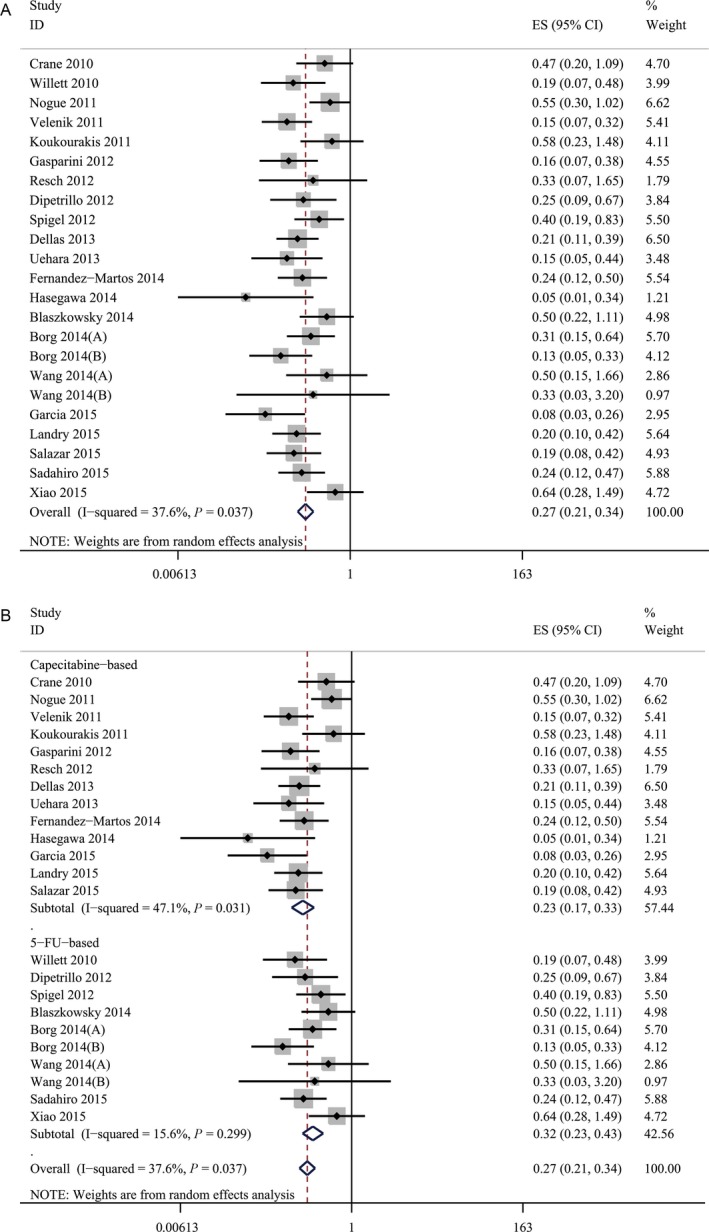
(A) The pooled estimate of pCR for bevacizumab‐relevant cohorts. (B) The results of subgroup analysis of bevacizumab‐relevant cohorts. The pooled estimates of pCR. pCR, pathologic complete response.

**Figure 3:**
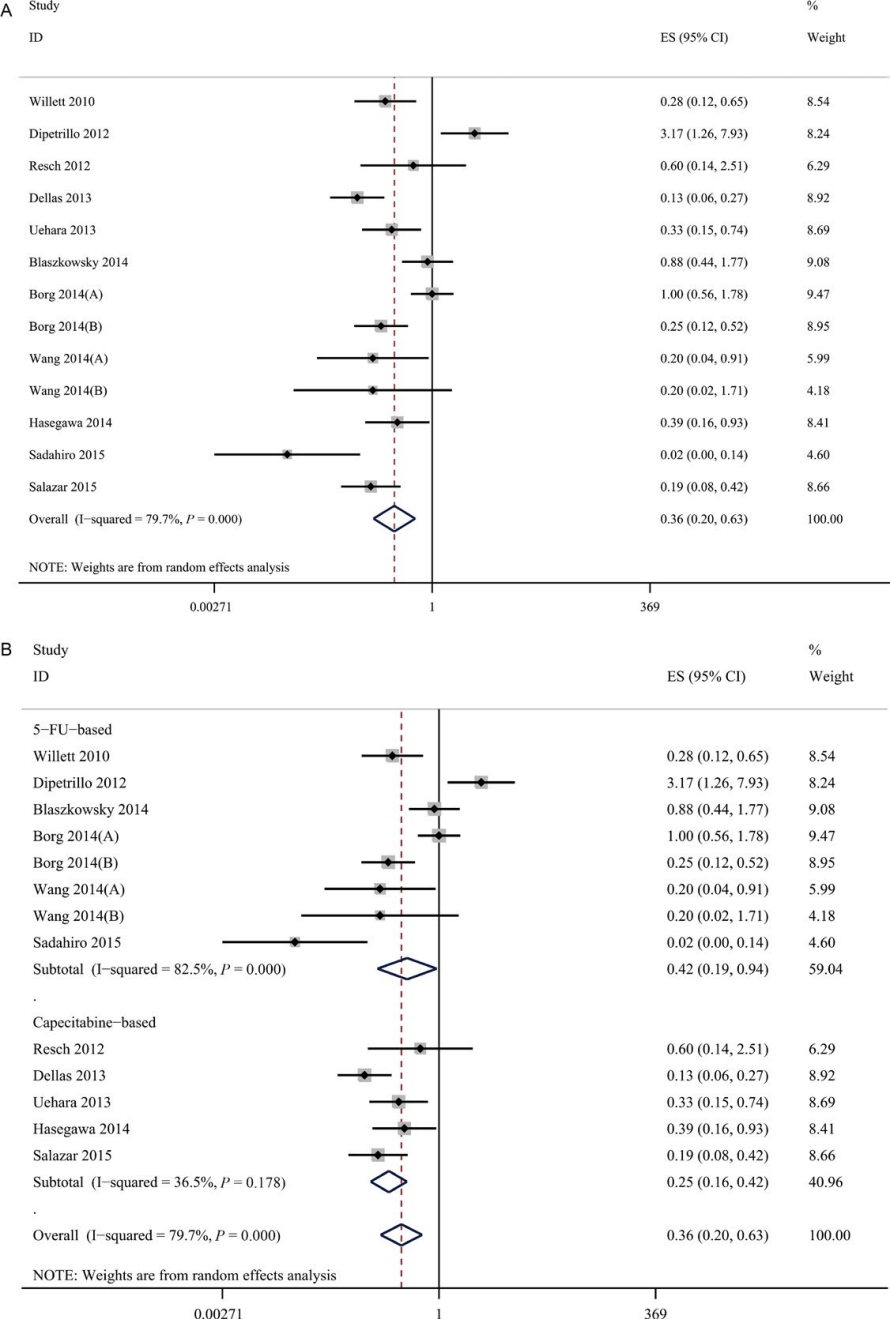
(A) The pooled estimate of preoperative Grade 3/4 toxicity for bevacizumab‐relevant cohorts. (B) The results of subgroup analysis of bevacizumab‐relevant cohorts. The pooled estimates of preoperative Grade 3/4 toxicity.

The pooled estimate of pCR for bevacizumab‐relevant cohorts was 27% (95%CI, 21–34%) (Fig. 2A). Meanwhile, the pooled estimate of preoperative Grade 3/4 toxicity for bevacizumab‐relevant cohorts was 36% (95% CI, 20–63%) (Fig. 3A). To better learn about the increased risk of clinically relevant toxicities, we listed the incidences of anti‐VEGF‐relevant toxicity focusing on bleeding, gastrointestinal perforation, and wound‐healing complication (shown in Table 4). The pooled estimates of Grade 3/4 bleeding, Grade 3/4 gastrointestinal perforation, and Grade 3/4 wound‐healing complication were also calculated and the results were 2.1% (95% CI, 1.0–4.7%) for Grade 3/4 bleeding, 1.9% (95% CI, 0.7–5.4%) for Grade 3/4 gastrointestinal perforation and 2.4% (95% CI, 1.0–6.2%) for Grade 3/4 wound‐healing complication.

**Table 4:** The treatment‐related toxicity status of patients who received additional anti‐VEGF or anti‐EGFR agents in neoadjuvant treatment

Study	Enrollment, n	Neoadjuvant therapy	Grade 3/4 treatment‐related toxicity^a^
Blaszkowsky 2014	32	5‐FU + Erlotinib + bevacizumab + RT	NR
Borg 2014	46	Folfox‐4 + bevacizumab	Grade 3/4 gastrointestinal perforation: 1/46 (2.17%)Grade 3/4 bleeding/hemorrhage: 2/46 (4.35%)Grade 3/4 wound‐healing complication: 0
	45	5‐FU + bevacizumab + RT	Grade 3/4 gastrointestinal perforation: 0Grade 3/4 bleeding/hemorrhage: 0Grade 3/4 wound‐healing complication: 2/45 (4.44%)
Crane 2010	25	Capecitabine + bevacizumab + RT	NR
Dellas 2013	69	Capox + bevacizumab + RT	Grade 3/4 delayed wound‐healing: 1/69 (1.45%)
Dipetrillo2012	25	mFOLFOX6 + bevacizumab + RT	Grade 3/4 bleeding: 1/25 (4%)
Fernandez‐Martos 2014	46	Capox + bevacizumab	NR
Garcia 2015	41	Capecitabine + bevacizumab + RT	NR
Gasparini 2012	43	Capecitabine + bevacizumab + RT	Grade 3/4 rectal hemorrhage: 0
Hasegawa 2014	25	Capox + bevacizumab	NR
Landry 2015	54	Capox + bevacizumab + RT	Grade 3/4 CNS hemorrhage: 1/54 (1.85%)
Nogue 2011	47	Capox + bevacizumab + RT	Grade 3/4 hemorrhage: 0
Resch 2012	8	Capecitabine + bevacizumab + RT	NR
Sadahiro 2015	52	S‐1 + bevacizumab + RT	NR
Spigel 2012	35	5‐FU + bevacizumab + RT	Grade 3/4 wound complication: 0
Uehara 2013	32	Capox + bevacizumab	Grade 3/4 perforation: 1/32 (3.13%)
Velenik 2011	61	Capecitabine + bevacizumab + RT	Grade 3/4 bleeding: 10/61 (16.39%)
Wang 2014	12	FOLFOX + bevacizumab + RT/5‐FU + bevacizumab + RT	NR
	6	FOLFOX + bevacizumab + RT	NR
Xiao 2015	25	5‐FU + oxaliplatin + bevacizumab + RT	NR
Koukourakis 2011	19	Capecitabine + bevacizumab + RT	NR
Salazar 2015	44	Capecitabine + bevacizumab + RT	NR
Willett 2010	32	5‐FU + bevacizumab + RT	NR
Bengala 2009	40	5‐FU + cetuximab + RT	NR
Horisberger 2009	50	Capecitabine + Irinotecan + cetuximab + RT	NR
Kim, S. Y 2011	40	CapIri + cetuximab + RT	Grade 3/4 diarrhea: 2/40 (12.5%)Grade 3/4 hand‐foot syndrome: 0Grade 3/4 skin rash: 2/40 (5%)
Machiels 2007	40	Capecitabine + cetuximab + RT	Grade 3/4 diarrhea: 6/40(15%);Grade 3/4 hand‐foot syndrome: 1/40 (2.5%);Grade 3/4 acneiform rash: 0
Rodel 2008	60	Capox + cetuximab + RT	Grade 3/4 diarrhea: 9/60 (15%)Grade 3/4 hand‐foot syndrome: 0Grade 3/4 radiation dermatitis: Grade 3: 4/60 (6.67%);Grade 3/4 acneiform rash: 2/60 (3.33%)
Sun 2012	63	Capecitabine + cetuximab + RT	Grade 3/4 diarrhea: 0Grade 3/4 hand and foot syndrome: 0Grade 3/4 radiodermatitis: 10/63 (15.87%)Grade 3/4 acneiform rash: 4/63 (6.35%)
Velenik 2012	47	Capecitabine + cetuximab + RT	Grade 3/4 diarrhea: 4/47 (8.51%)Grade 3/4 hand‐foot syndrome: 0Grade 3/4 acneiform rash: 0
Dewdney 2012	83	Capecitabine + cetuximab + RT	NR
Jin 2015	21	Capecitabine + nimotuzumab + RT	Grade 3/4 diarrhea: 2/21 (9.52%)Grade 3/4 hand‐foot skin reaction: 0Grade 3/4 radiation dermatitis: 0Grade 3/4 acneiform rash: 0
Helbling 2013	40	Capecitabine + panitumumab + RT	Grade 3/4 diarrhea: 4/40 (10%)Grade 3/4 hand‐foot syndrome: 1/40 (2.5%)Grade 3/4 acneiform rash: 1/40 (2.5%)
Pinto 2011	60	5‐FU + oxaliplatin + panitumumab + RT	Grade 3/4 diarrhea: 23/60 (38.33%)Grade 3/4 hand‐foot syndrome: 0Grade 3/4 acneiform rash: 11/60 (18.33%)

RT, radiotherapy; 5‐FU, fluorouracil; FOLFOX, leucovorin plus fluorouracil plus oxaliplatin; Capox, capecitabine plus oxaliplatin; S‐1, tegafur plus gimeracil plus potassium oxonate; NR, not reported.

a We focused on bleeding and bowel perforation and impaired wound‐healing for anti‐VEGF‐relevant cohorts and diarrhea and skin changes in the affected area of the skin involved in radiotherapy for anti‐EGFR‐relevant cohorts.

To further evaluate the efficacy and safety of bevacizumab, we performed a subgroup analysis by separating the bevacizumab‐relevant cohorts into two subgroups: the 5‐fluorouracil‐based (5‐FU‐based) bevacizumab group and the capecitabine‐based bevacizumab group. The results of the subgroup analysis showed that the 5‐FU‐based bevacizumab group had a pooled estimate for pCR of 32% (95% CI, 23–43%) (Fig. 2B) and the pooled estimate of preoperative Grade 3/4 toxicity reached 42% (95% CI, 19–94%) (Fig. 3B). For capecitabine‐based bevacizumab group, a pooled pCR of 23% (95% CI, 17–33%) was achieved (Fig. 2B) along with a pooled estimate of preoperative Grade 3/4 toxicity of 25% (95% CI, 16–42%) (Fig. 3B).

### The efficacy and safety of EGFR inhibitors

**Figure 4:**
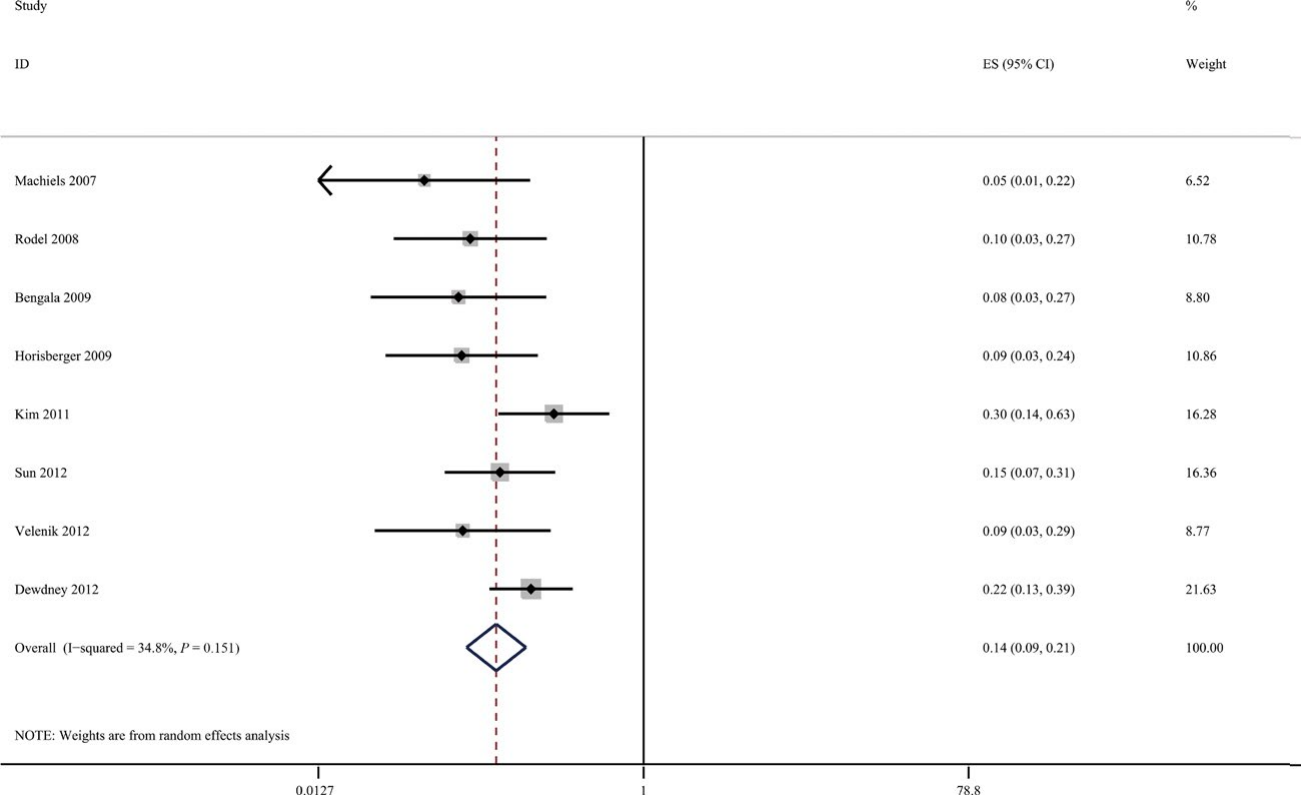
The pooled estimate of pathologic complete response for cetuximab‐relevant cohorts.

The pooled estimate of pCR for cetuximab‐relevant studies was 14% (95% CI, 9–21%) (Fig. 4). One study 58 reported a preoperative Grade 3/4 toxicity of approximately 17.9%, while the others did not report toxicity in this manner.

We reviewed the few studies involving the other EGFR inhibitors, although their low numbers made additional analysis unavailable. In the only study 63 focusing on nimotuzumab, four (19%) of 21 enrolled patients achieved pCR. For the two studies studying panitumumab, one 65 reported a pCR rate of 21.1% in 57 eligible patients and the other, a RCT 64 showed a 10% pCR rate for patients receiving panitumumab in addition with nCRT versus 18% for patients treated with nCRT alone.

To comprehensively evaluate the increased risk of clinically relevant toxicities, we listed the incidences of anti‐EGFR‐relevant toxicity focusing on diarrhea and skin changes in the affected area of the skin involved in radiotherapy (shown in Table 4). The pooled estimates of Grade 3/4 diarrhea, Grade 3/4 hand‐foot syndrome, Grade 3/4 rash, and Grade 3/4 radiodermatitis were also calculated and the results were 13.3% (95% CI, 6.4–27.9%) for Grade 3/4 diarrhea, 1.5% (95% CI, 0.6–3.7%) for Grade 3/4 hand‐foot syndrome, 5.2% (95% CI, 2.2–11.9%) for Grade 3/4 rash and 10.7% (95% CI, 4.2–27.1%) for Grade 3/4 radiodermatitis.

### Evaluation of publication bias

To evaluate publication bias, we performed Begg's test and Egger's test. The *P* values of Begg's test and Egger's test for the pooled pCR of bevacizumab‐relevant cohorts were 0.303 and 0.277 (Fig. S2). The *P* values of Begg's test and Egger's test for the pooled preoperative Grade 3/4 toxicity of bevacizumab‐relevant cohorts were 0.714 and 0.257 (Fig. S3). The *P* values of Begg's test and Egger's test for the pooled pCR of cetuximab‐relevant cohorts were 0.048 and 0.005 (Fig. S4). To further evaluate the potential publication bias detected from the pooled pCR of cetuximab‐relevant cohorts, we performed sensitivity analysis, the results are shown in Figure S5.

## Discussion

Since the use of neoadjuvant therapies began, a tremendous amount of work has been done to improve the regimens. Abundant clinical trials and two meta‐analyses have revealed the efficacy of preoperative radiotherapy granting better local control and a lower rate of local recurrence for LARC patients compared with surgery alone 66, 67. Subsequently, the addition of 5‐FU or capecitabine to neoadjuvant radiotherapy was demonstrated to significantly increase the incidence of pCR, and they have been widely accepted as first‐line anticancer regimens in the clinic 8, 68. More recently, researchers have studied the roles of various targeted agents added to the nCRT setting in pursuit of higher pCR rates for LARC patients. However, whether or not the addition of targeted agents to the nCRT regimens provides increased efficacy remains controversial and requires further investigation.

Until now, there have been limited RCTs and CCTs investigating the roles of targeted agents in nCRT regimens for LARC patients, and most of the studies in this field were single‐arm phase II studies. These single‐arm phase II studies basically focus on the pCR rates to demonstrate the efficacy of a certain targeted agent, and often lack data regarding patient survival status 35, 46, 47. Under these circumstances, a benchmark pCR rate would be necessary to be able to evaluate the efficacy of the additional targeted agents to the nCRT regimens. However, single‐arm phase II clinical trials lack a putative benchmark and usually evaluate the efficacy by comparing their pCR results with their predefined goal for pCR rate or the results of pCR in other studies 21, 22, 23. To help evaluate the efficacy of bevacizumab when added to the neoadjuvant therapy for LARC patients, we established a benchmark by quantitatively synthesizing the pCR rates of neoadjuvant therapy regimens without added targeted agents for LARC patients. We extracted pCR rates from ten cohorts that met our patient enrollment criteria and without any targeted agents in their nCRT regimens from the pooled analysis of Maas et al. 26. The baseline characteristics of these cohorts are shown in Table S1 and the pooled estimate of pCR of these cohorts was 17% (95% CI, 15–20%) (Fig. S1). This benchmark is also in the range of the pCR rates reported in several other previous studies 3, 4, 8, 10, 11, 12. Therefore, we believe that 17% is an adequate benchmark that can help reasonably evaluate the efficacy of adding targeted agents to the nCRT for LARC patients.

Willett et al. 69 were the pioneers in investigating the role of bevacizumab in 5‐FU‐based nCRT, and they achieved a feasible pCR rate of 16%. Other researchers also devoted themselves to evaluating the efficacy of bevacizumab in nCRT for LARC patients 21, 22, 23. In our study, we achieved a pooled pCR rate (27%) over the benchmark (17%) and thus, demonstrated an appreciable pCR for the addition of bevacizumab to neoadjuvant therapy for LARC patients. Moreover, the results of the subgroup analysis showed that the 5‐FU‐based group achieved a higher pooled estimate of pCR (32%) than capecitabine‐based group (23%), yet the pCR rates for both groups were higher than the benchmark (17%). One previous study demonstrated 70 that capecitabine‐based nCRT was superior to 5‐FU‐based nCRT in 5‐year overall survival, 3‐year DFS, reduction in distant metastasis, and pCR rate. Alternatively, a more recent meta‐analysis comparing the efficacies of oral capecitabine and infusional 5‐FU 71 demonstrated no significant difference between the pCR rates of the two groups in a neoadjuvant setting. The NCCN guidelines also comment that the efficacy of these two drugs is “equivalent” 7. In pursuit of a plausible explanation, we extracted and evaluated the RT status of bevacizumab‐relevant cohorts considering the tumor‐downsizing nature of RT (shown in Table S2). Three of the 13 capecitabine‐based cohorts do not include RT in their neoadjuvant therapy while only one of the 10 5‐FU‐based cohorts does not include RT. And the pCR of two of these three capecitabine‐based cohorts are distinctly low, merely 4.3% (1/23) and 13.3% (4/30). Besides, a total of 53 individuals who did not receive RT hold over a tenth of the whole capecitabine‐based group population. These may help explain the controversial result of this subgroup analysis to some extent. In summary, bevacizumab shows appreciable efficacy in nCRT for LARC patients, and this efficacy is consistent in 5‐FU‐based nCRT and capecitabine‐based nCRT. As our enrolled studies are mostly phase II clinical trials, this efficacy can encourage more incoming phase III clinical trials and serve as evidence of a promising outlook for future clinical applications of bevacizumab in nCRT regimens for LARC patients.

It is well‐known that chemotherapy can cause toxicity in patients. Thus, it is inevitable that adding targeted agents in nCRT regimens could result in extra toxicity. Sauer et al. 4 reported a Grade 3/4 toxicity rate of 27% in 399 rectal cancer patients receiving preoperative chemoradiotherapy. Two other important RCTs 3, 8 showed that Grade 3/4 toxicity occurred in 13.9% and 14.6% of their enrolled patients, respectively, in the duration of nCRT. A previous study 72 also demonstrated that LARC patients in two cohorts with different nCRT regimens without any targeted agents reached pCR rates of 17% and 13% at the cost of Grade 3/4 toxicities of 23% and 20%, respectively. In our study, the pooled estimates of preoperative Grade 3/4 toxicity (36% for total bevacizumab‐relevant cohorts, 42% for 5‐FU‐based bevacizumab group cohorts, and 25% for capecitabine‐based bevacizumab group cohorts) are reasonable considering the high rates of pooled pCR (27%, 32%, and 23%, respectively) in bevacizumab‐relevant cohorts. Additionally, the incidences of anti‐VEGF‐relevant toxicity listed in Table 4 and the pooled estimates indicate that anti‐VEGF treatment‐relevant toxicities are relatively mild. Thus, we presume that the safety of bevacizumab is acceptable.

The role of cetuximab, an anti‐EGFR monoclonal antibody, in nCRT for LARC patients has been investigated by many researchers in recent years 56, 59. In our study, we found that the pooled estimate for pCR in cetuximab‐relevant cohorts is less than the benchmark, which may indicate an inadequate efficacy of adding cetuximab to the nCRT for LARC patients. Increasing evidences have demonstrated that KRAS‐mutated patients cannot benefit from anti‐EGFR treatments 34, 73, 74, 75. It is also a well‐known fact that anti‐EGFR activity might be also strictly dependent on the presence/lack of mutations in NRAS or BRAF genes 7, 76, 77. In our study, most of the included cetuximab‐relevant studies only focus on KRAS status and did not report their pCR rates according to the KRAS status of the enrolled patients. Thus, the inadequate pooled pCR rate of cetuximab‐relevant cohorts may be due to the lack of published mutation status. As such, additional investigations are needed to explore the efficacy of adding cetuximab to the neoadjuvant therapy specifically for RAS and BRAF wild‐type LARC patients.

The few studies 63, 64, 65 investigating the roles of nimotuzumab and panitumumab in the nCRT for LARC patients did not show convincing evidence for efficacy or safety, so more investigations regarding nimotuzumab and panitumumab are urgently needed. Two ongoing surveys 78, 79 focusing on bevacizumab and lapatinib are expected to provide more evidence on the outcome of LARC patients in a couple of years.

Despite the inadequate pCR, the addition of anti‐EGFR agents presents acceptable safety and this safety may facilitate more anti‐EGFR‐oriented clinical trials.

No publication bias was detected in the meta‐analysis for bevacizumab‐relevant cohorts. However, the results of Begg's and Egger's tests concerning the pCR for cetuximab‐relevant cohorts suggested the existence of potential publication bias. The results of sensitivity analysis, as shown in Figure S5, seem to indicate that the pooled pCR of cetuximab‐relevant cohorts deviates from the current value most when Dewdney et al's study is omitted. Thus, we comprehensively reviewed this well‐designed RCT of Dewdney et al's and found that the pCR rate of their cetuximab‐relevant arm (18%) was higher than most of the other included cetuximab‐relevant cohorts. Meanwhile, this cohort held the largest weight in the quantitative analysis due to the largest sample size (83) among all of the inclusions. Besides, over half of the population (46) in this cohort are KRAS/BRAF wild type which is previously reported to present good response to anti‐EGFR treatment. All of the above accounts for the higher pCR presented in this cohort and explains why this pCR influences the pooled estimate most.

Our study is the first meta‐analysis to evaluate the efficacy of targeted agents in the nCRT for LARC patients. However, several limitations exist in our study. First, due to the lack of relevant RCTs and CCTs, we conducted this meta‐analysis in a single‐arm setting. Second, we only focused on pCR and its indicative role in our meta‐analysis and we lack data regarding perioperative and postoperative outcomes including operation time, perioperative complication rate, and postoperative recovery time so that we cannot directly evaluate the potential influences that adding targeted agents may have on the following curative surgical resection and postoperative recovery of LARC patients which highly concern clinical practitioners in this field. Third, cohort numbers from single‐arm studies included in this study are mostly small‐scale, which can lead to over‐reporting of the efficacy of these neoadjuvant regimens. Meanwhile, heterogeneity is, to an extent, inevitable among these multi‐center studies. Fourth, most of the anti‐EGFR cohorts are small‐scale and stress KRAS status only. To the best of our knowledge, however, anti‐EGFR activity might be also strictly determined by the mutational statuses of NRAS and BRAF. Fifth, when conducting this study, we only focused on published studies and extracted data available in the text, thus, we did not have access to relevant individual patient data, which could help us improve the analysis of the treatment effects of the targeted agents. Despite these limitations, we found that there is increased efficacy when adding bevacizumab to nCRT for LARC patients.

## Conclusion

In conclusion, our study revealed that adding bevacizumab to the neoadjuvant therapy regimens provides an appreciable pCR for LARC patients. However, more RCTs are needed for further validation. Meanwhile, the efficacy of cetuximab remains inconclusive, RCTs with larger scale and better study design that stress more on mutational status are needed.

## Conflict of Interest

The authors declare that they have no conflict of interest.

## Supporting information


**Figure S1.** The establishment of benchmark for pCR.

**Figure S2.** (a) The Begg's funnel plots concerning the pCR for bevacizumab‐relevant studies. (b) The Egger's publication bias plot concerning the pCR for bevacizumab‐relevant studies.

**Figure S3.** (a) The Begg's funnel plots concerning the preoperative Grade 3/4 toxicity for bevacizumab‐relevant studies. (b) The Egger's publication bias plot concerning the preoperative Grade 3/4 toxicity for bevacizumab‐relevant studies.

**Figure S4**. (a) The Begg's funnel plots concerning the pCR for cetuximab‐relevant studies. (b) The Egger's publication bias plot concerning the pCR for cetuximab‐relevant studies.

**Figure S5.** The results of the sensitivity analysis concerning the pCR for cetuximab‐relevant studies.

**Table S1.** Data from Maas, et al's pooled analysis.

**Table S2.** The radiotherapy status of Bevacizumab‐relevant cohorts.
