# Supplementary figures and images for: The efficacy of adding targeted agents to neoadjuvant therapy for locally advanced rectal cancer patients: a meta‐analysis
Source: Cancer Med. 2018 Feb 21;7(3):565–82. doi: 10.1002/cam4.1298 (PMC5852374; doi:10.1002/cam4.1298)

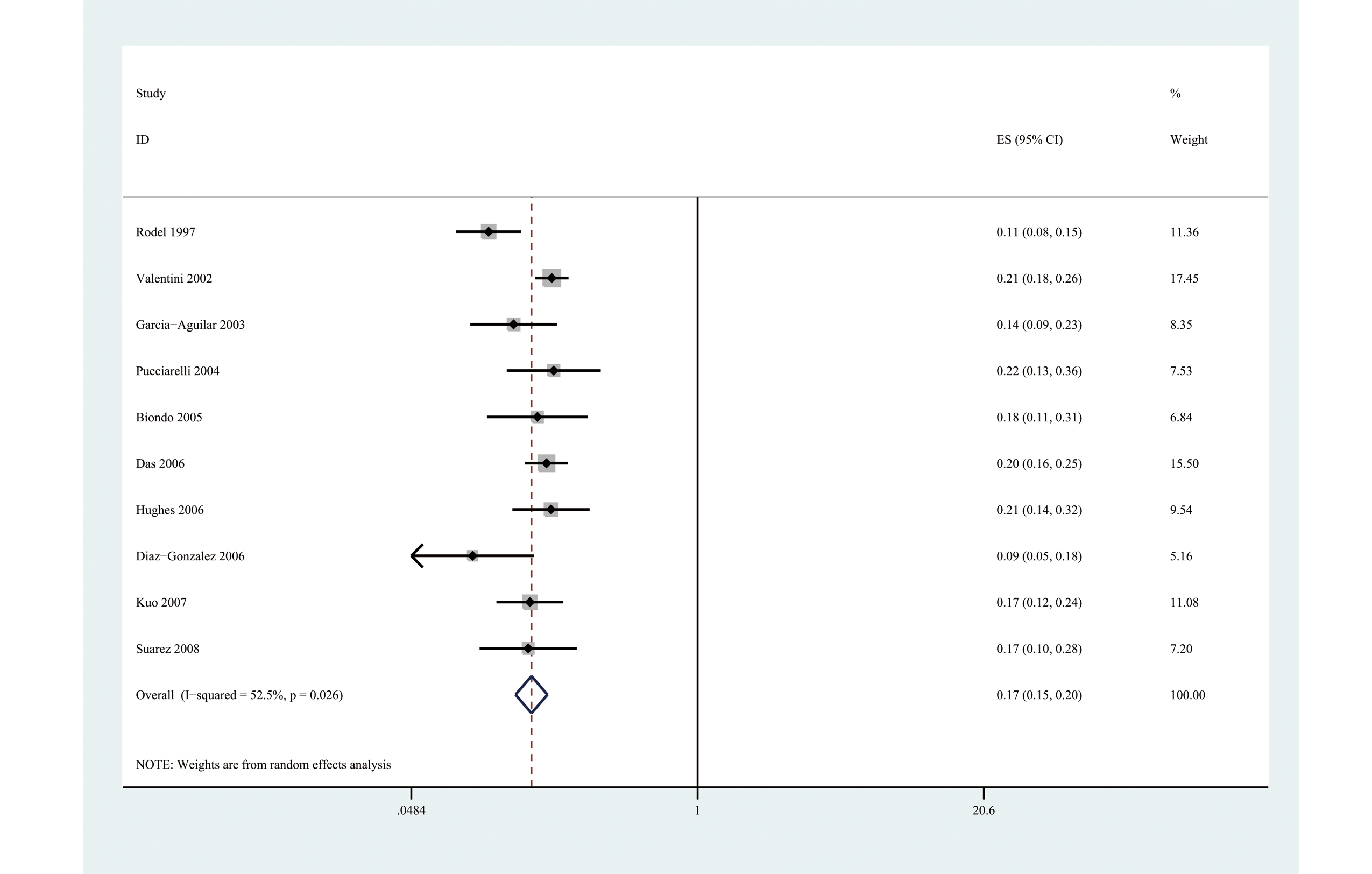

Supplement: Supplementary file 1 — Figure S1. The establishment of benchmark for pCR. [file CAM4-7-565-s001.tif]

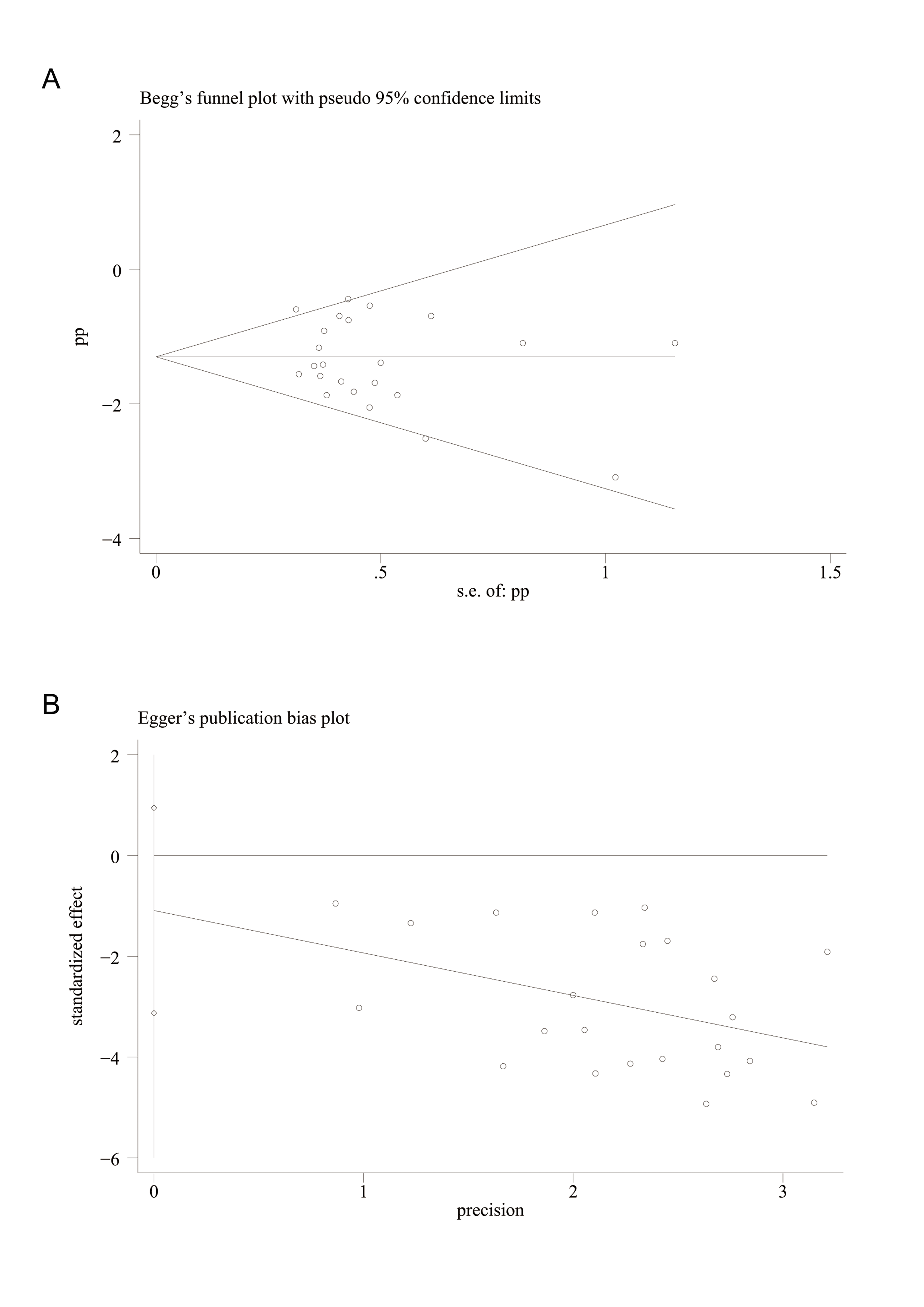

Supplement: Supplementary file 2 — Figure S2. (a) The Begg's funnel plots concerning the pCR for bevacizumab‐relevant studies. (b) The Egger's publication bias plot concerning the pCR for bevacizumab‐relevant studies. [file CAM4-7-565-s002.tif]

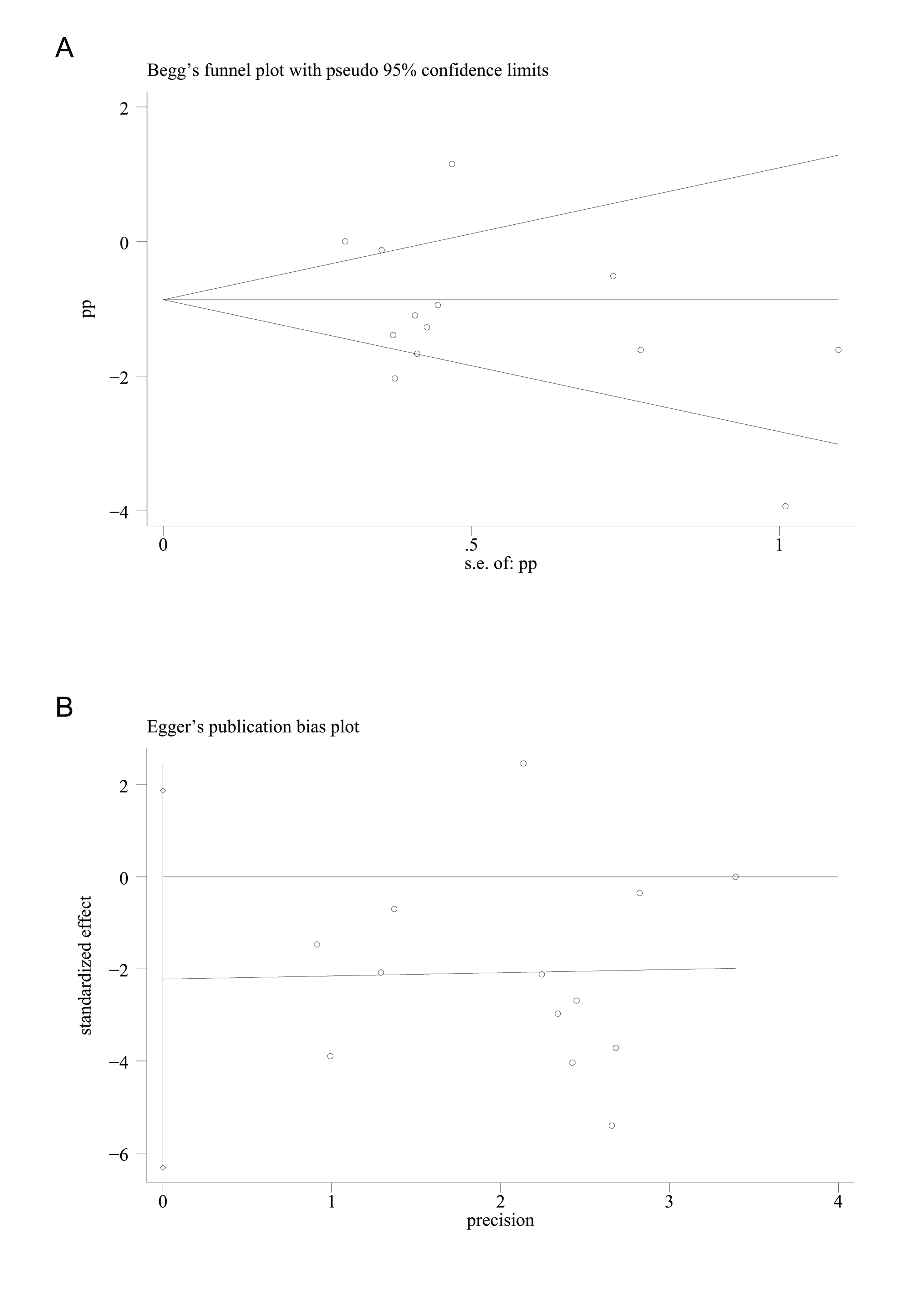

Supplement: Supplementary file 3 — Figure S3. (a) The Begg's funnel plots concerning the preoperative Grade 3/4 toxicity for bevacizumab‐relevant studies. (b) The Egger's publication bias plot concerning the preoperative Grade 3/4 toxicity for bevacizumab‐relevant studies. [file CAM4-7-565-s003.tif]

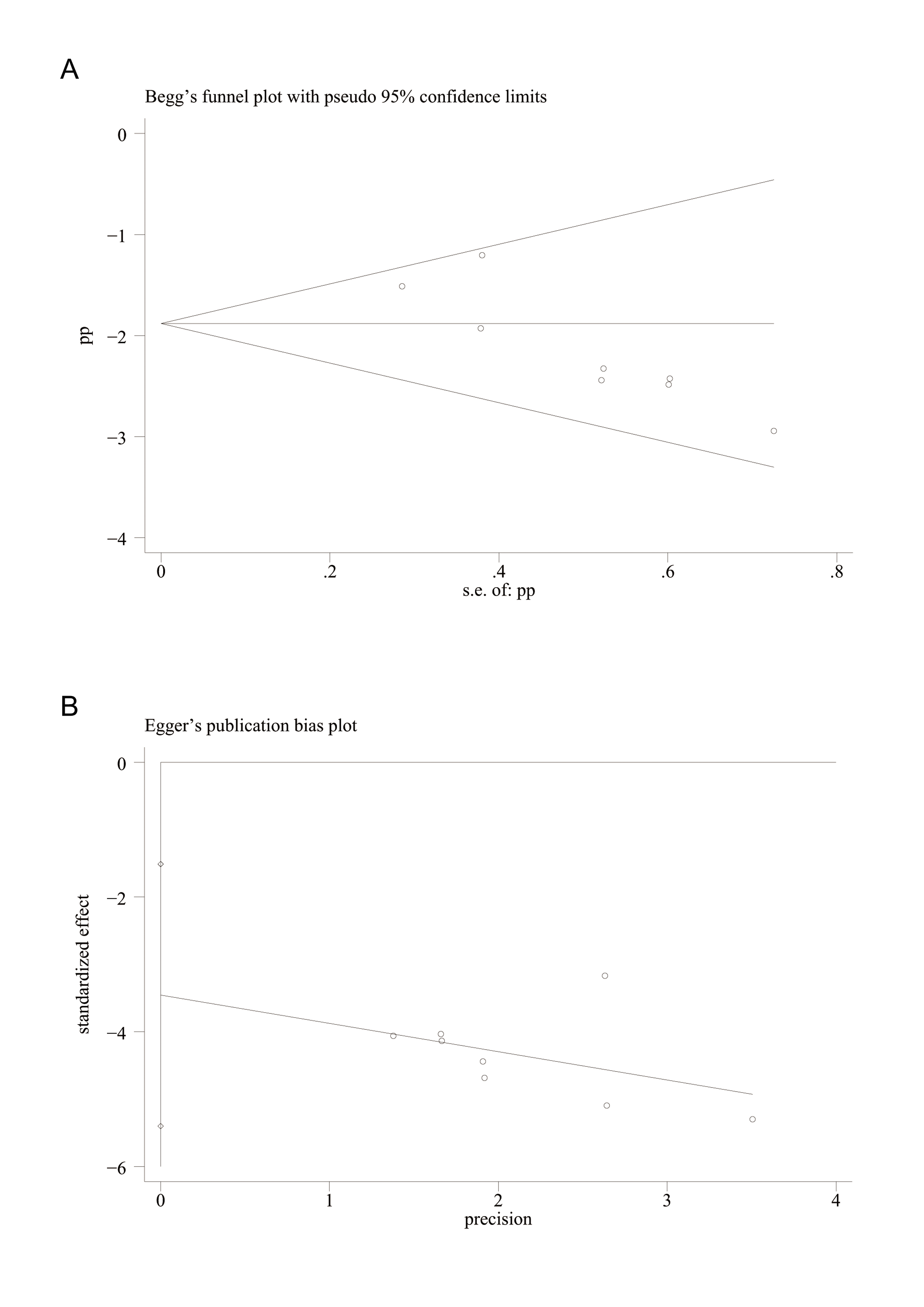

Supplement: Supplementary file 4 — Figure S4. (a) The Begg's funnel plots concerning the pCR for cetuximab‐relevant studies. (b) The Egger's publication bias plot concerning the pCR for cetuximab‐relevant studies. [file CAM4-7-565-s004.tif]

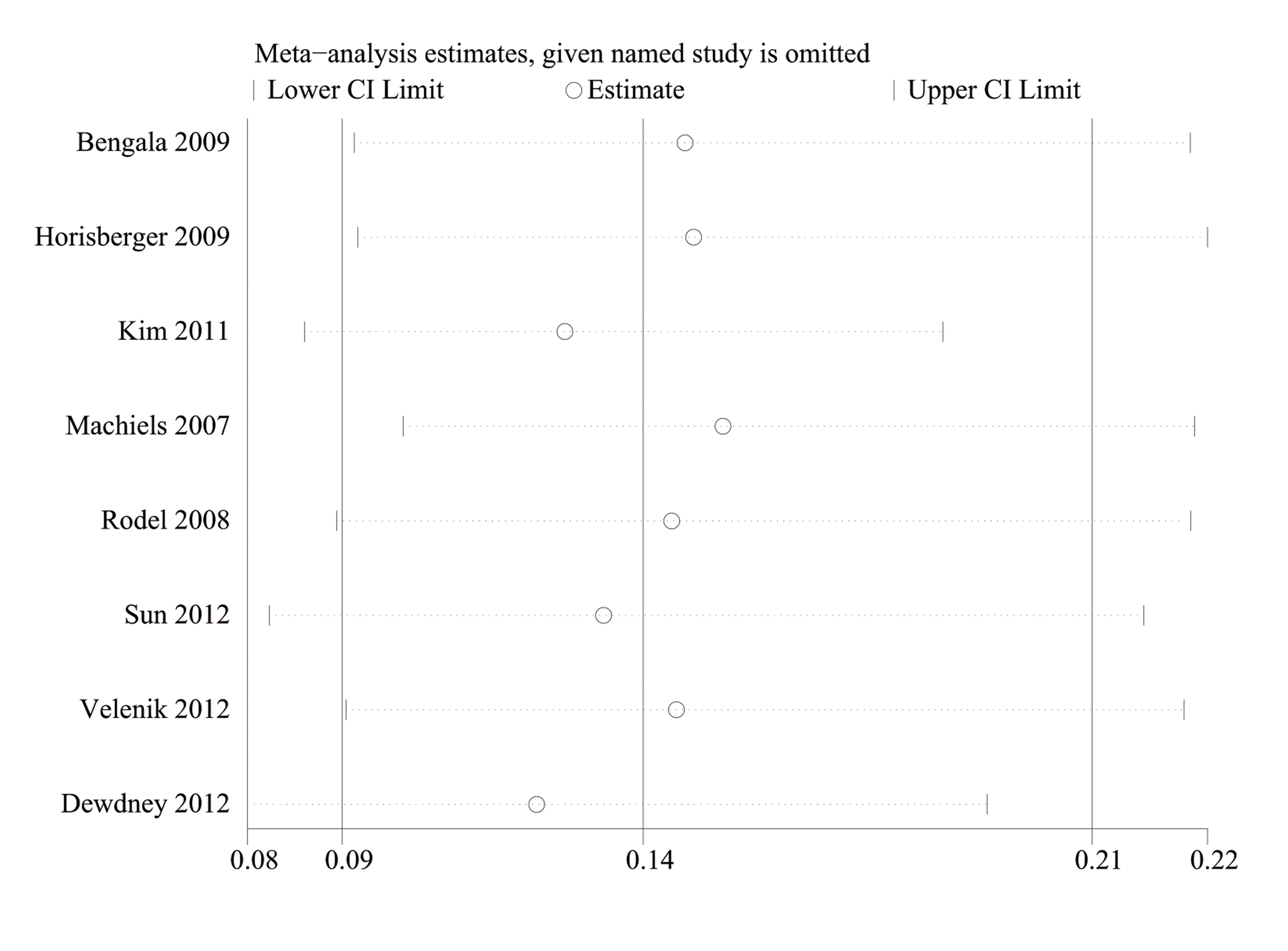

Supplement: Supplementary file 5 — Figure S5. The results of the sensitivity analysis concerning the pCR for cetuximab‐relevant studies. [file CAM4-7-565-s005.tif]
