# Supplementary material for: The efficacy of adding targeted agents to neoadjuvant therapy for locally advanced rectal cancer patients: a meta‐analysis
Source: Cancer Med. 2018 Feb 21;7(3):565–82. doi: 10.1002/cam4.1298 (PMC5852374; doi:10.1002/cam4.1298)
Supplement: Supplementary file 6 — Table S1. Data from Maas, et al's pooled analysis. [file CAM4-7-565-s006.docx]

**Supporting Information Table 1. Data from Maas, M. et al’s pooled analysis**

| **Study** | **Population** | **Type of neoadjuvant therapy** | **pCR** |
| --- | --- | --- | --- |
| Valentini 2002 | LARC,T3-4 or N+ | External RT +/– IORT, FU+MITOC or CIS | 17.6%  (152/862) |
| Das 2006 | LARC, M0 | External RT, FU or TEG with uracil | 16.8%  (90/535) |
| Rodel 1997 | Stage II–III | External RT, FU | 9.9%  (36/364) |
| Kuo 2007 | T3–4, N+, M0 | External RT, FU, MITOC | 14.5%  (36/248) |
| Garcia-Aguilar 2003 | Stage II–III | External RT, FU | 12.5%  (21/168) |
| Hughes 2006 | T3–4 | External RT, FU | 17.5%  (28/160) |
| Diaz-Gonzalez 2006 | T3–4, N+ | External RT+IORT, FU or TEG | 8.5%  (10/118) |
| Suarez 2008 | LARC | External RT, FU | 14.3%  (17/119) |
| Pucciarelli 2004 | T3–4, N+, M0 | External RT, FU+LV or CARBO or OX | 17.9%  (19/106) |
| Biondo 2005 | T3–4, LARC | External RT, FU | 15.5%  (16/103) |

Abbreviations: LARC= locally advanced rectal cancer. N+= positive clinical nodal status. RT= radiotherapy. IORT= intraoperative radiotherapy. FU= fluorouracil. MITOC= mitomycin C.

CIS= cisplatin. M0= no signs of distant metastases. TEG= tegafur. LV= leucovorin. CARBO= carboplatin. OX= oxaliplatin.
