# Supplementary material for: The efficacy of adding targeted agents to neoadjuvant therapy for locally advanced rectal cancer patients: a meta‐analysis
Source: Cancer Med. 2018 Feb 21;7(3):565–82. doi: 10.1002/cam4.1298 (PMC5852374; doi:10.1002/cam4.1298)
Supplement: Supplementary file 7 — Table S2. The radiotherapy status of Bevacizumab‐relevant cohorts. [file CAM4-7-565-s007.docx]

**Supporting Information Table S2. The radiotherapy status of Bevacizumab-relevant cohorts**

| Study | Neoadjuvant therapy | †RT status | pCR |
| --- | --- | --- | --- |
| Blaszkowsky 2014 | 5-FU+Erlotinib+bevacizumab+ RT | Patients received 45 Gy in 1.8 Gy/fraction to the initial field and 5.4 Gy in 1.8 Gy/fraction to the boost field to a total dose of 50.4 Gy | 33.3%  (9/27) |
| Borg 2014 | Folfox-4+bevacizumab | No RT | 23.8%  (10/42) |
|  | 5-FU+bevacizumab+ RT | The total dose of RT was 45 Gy delivered in 25 fractions of 1.8 Gy, 5 days/week. | 11.4%  (5/44) |
| Crane 2010 | Capecitabine+bevacizumab+ RT | Patients received 45 Gy in 1.8 Gy/fraction to the initial field and 5.4 Gy in 1.8 Gy/fraction to the boost field to a total dose of 50.4 Gy | 32%  (8/25) |
| Dellas 2013 | Capox+bevacizumab+ RT | All patients received a total dose of 50.4 Gy, with daily fractions of 1.8 Gy on 5 days per week. | 17.4%  (12/69) |
| Dipetrillo2012 | mFOLFOX6+bevacizumab+ RT | Patients received 45 Gy in 1.8 Gy/fraction to the initial field and 5.4 Gy in 1.8 Gy/fraction to the boost field to a total dose of 50.4 Gy | 20%  (5/25) |
| Fernandez-Martos 2014 | Capox+ bevacizumab | No RT | 19.6%  (9/46) |
| Garcia 2015 | Capecitabine+bevacizumab+ RT | The total dose of RT was 45 Gy delivered in 25 fractions of 1.8 Gy, 5 days/week. | 7.5%  (3/40) |
| Gasparini 2012 | Capecitabine+bevacizumab+ RT | Patients received 45 Gy in 1.8 Gy/fraction to the initial field and 5.4 Gy in 1.8 Gy/fraction to the boost field to a total dose of 50.4 Gy | 14.0%  (6/43) |
| Hasegawa 2014 | Capox+bevacizumab | No RT | 4.3%  (1/23) |
| Landry 2015 | Capox+bevacizumab+RT | Patients received 45 Gy in 1.8 Gy/fraction to the initial field and 5.4 Gy in 1.8 Gy/fraction to the boost field to a total dose of 50.4 Gy | 17.0%  (9/53) |
| Nogue 2011 | Capox+bevacizumab+RT | RT consisted of a total of 45 Gy delivered in 25 daily fractions over 5 weeks (1.8 Gy/d for 5 d/wk), followed by a boost of 5.4 Gy (1.8 Gy/d for 3 days) | 35.6%  (16/45) |
| Resch 2012 | Capecitabine+bevacizumab+ RT | The total dose of RT was 45 Gy delivered in 25 fractions of 1.8 Gy, 5 days/week. | 25%  (2/8) |
| Sadahiro 2015 | S-1+bevacizumab+ RT | The total dose of RT was 45 Gy delivered in 25 fractions of 1.8 Gy, 5 days/week. | 19.2%  (10/52) |
| Spigel 2012 | 5-FU+bevacizumab+ RT | The total dose of RT was 50.4 Gy delivered in 28 fractions of 1.8 Gy, 5 days/week. | 28.6%  (10/35) |
| Uehara 2013 | Capox+bevacizumab | No RT | 13.3%  (4/30) |
| Velenik 2011 | Capecitabine+bevacizumab+ RT | Patients received 45 Gy in 1.8 Gy/fraction to the initial field and 5.4 Gy in 1.8 Gy/fraction to the boost field to a total dose of 50.4 Gy | 13.3%  (8/60) |
| Wang 2014 | FOLFOX+bevacizumab+ RT/5-FU+bevacizumab+ RT | The total dose of RT was 45 Gy delivered in 25 fractions of 1.8 Gy, 5 days/week. | 33.3%  (4/12) |
|  | FOLFOX+bevacizumab+ RT | The total dose of RT was 45 Gy delivered in 25 fractions of 1.8 Gy, 5 days/week. | 25%  (1/4) |
| Xiao 2015 | 5-FU+oxaliplatin+bevacizumab+ RT | RT was applied with a total dose of 50 Gy delivered in 25 fractions over 5 weeks (2.0 Gy for 5 day/week) | 39.1%  (9/23) |
| Koukourakis 2011 | Capecitabine+bevacizumab+ RT | A total dose of 34 Gy consisted of daily fraction of 3.4 Gy (5 fractions per week) for 9 consecutive fractions and a tenth fraction to the boost field (overall treatment time, 12 days) | 36.8%  (7/19) |
| Salazar 2015 | Capecitabine+bevacizumab+ RT | RT consisted of a total of 45 Gy delivered in 25 daily fractions over 5 weeks (1.8 Gy/day for 5 day/week) | 15.9%  (7/44) |
| Willett 2010 | 5-FU+bevacizumab+ RT | The total dose of RT was 50.4 Gy delivered in 28 fractions of 1.8 Gy, 5 days/week. | 15.6%  (5/32) |

†The techniques of RT in included studies were pretty much the same, thus we focused on the dosage of RT.

Abbreviations: RT: radiotherapy; Gy: gray; 5-FU: fluorouracil; FOLFOX: leucovorin plus fluorouracil plus oxaliplatin; Capox: capecitabine plus oxaliplatin; S-1: tegafur plus gimeracil plus potassium oxonate; NR: not reported;
